# Changes in the prevalence and trends of female genital mutilation in Iraqi Kurdistan Region between 2011 and 2018

**DOI:** 10.1186/s12905-021-01282-9

**Published:** 2021-04-01

**Authors:** Nazar P. Shabila

**Affiliations:** Department of Community Medicine, Hawler Medical University, Erbil, Iraq

**Keywords:** Female genital mutilation, Prevalence, Trend, Decline, Education

## Abstract

**Background:**

Female genital mutilation (FGM) is commonly practiced in Iraqi Kurdistan Region, where there are extensive efforts to combat the practice over the last decade. This study aimed to determine the trends and changes in the FGM prevalence in Iraq between 2011 and 2018 and assess their associated factors.

**Methods:**

Secondary data analysis of the Iraq Multiple Indicator Cluster Survey 2011 and 2018 was carried out to calculate the prevalence and the relative changes in the prevalence of FGM for 2011 and 2018 by governorate. The change in the prevalence was compared with the changes in other exposure variables such as age, education level, wealth, and area of residence over the same period.

**Results:**

The prevalence of FGM in 2018 was high in Erbil and Suleimaniya governorates (50.1% and 45.1%). The prevalence of FGM decreased remarkably from 2011 to 2018 in all governorates of the Iraqi Kurdistan Region. The decrease in the prevalence was statistically significant in Erbil and Suleimaniya. FGM prevalence declined remarkably in all age, education level, residence area groups, and most economic level groups. Such decline was associated with a significant increase in the education level, wealth, and urban residence. The decline was highest in the younger age groups, with a relative change of − 43.0% among 20–24 years and − 39.2% among 15–19 years. The decline was also highest in those with secondary and higher education (relative change = −32%). The decline was higher in rural areas than in urban areas (relative change = −35.3% and − 27.4%, respectively). The decline was higher among the poorest and second wealth quintile (relative change = −38.8% and − 27.2%, respectively).

**Conclusion:**

The trend of FGM in Iraqi Kurdistan Region declined remarkably and significantly from 2011 to 2018. Further decline is predicted because of having lower rates and a higher decline in the younger age groups. However, the rates remained high in Erbil and Suleimaniya governorates that need further intensifying the preventive measures. The education level of women plays a primary role in decreasing the prevalence and should be considered in future efforts to ban the practice.

## Background

Female genital mutilation (FGM), also called female genital cutting, involves the partial or total removal of external female genitalia or other injury to the female genital organs for non-medical reasons [1]. FGM is usually performed by a blade or shard of glass by an elder or a midwife with limited training and often under unhygienic conditions [2]. FGM is associated with different health consequences and even death. The practice can results in pain, bleeding, infection, and urinating problems as immediate health consequences. Chronic infections, cysts, chronic pain, birth complications, and sexual and emotional suffering are examples of long-term consequences of FGM [2–4].

FGM is considered a clear violation of women's and girls’ rights. It is also an extreme form of discrimination against girls and women. It is a violation of the children's rights since the victims are primarily young girls from infancy to 15 years. FGM undermines the girls' and women’s rights to health, physical integrity, and security. It also exposes girls and women to torture and inhuman or degrading treatment [5].

FGM is a deeply rooted tradition, which is practiced in 30 countries in Africa, Asia, and the Middle East. More than 200 million girls and women have been subjected to some forms of FGM in these 30 countries. Each year, more than three million girls younger than 15 years are at risk of FGM [5, 6].

FGM is increasingly being medicalized allegedly as a harm reduction strategy to reduce its negative health effects [7]. However, the medicalization of FGM does not have any benefits and will not reduce the long term consequences of FGM. There are no medical indications for FGM. The performance of FGM by health professionals violates the code of medical ethics. It can also result in a setback in the FGM eradication efforts [8].

### FGM in Iraqi Kurdistan Region

FGM is a common practice in Iraqi Kurdistan Region, an autonomous region in northern Iraq comprising the three governorates of Erbil, Suleimaniya, and Duhok. A prevalence of 40% was recorded in the early 2010s, which varied by the geographical areas and governorates from 4% in Dohuk to 58% in Erbil. Much higher rates were recorded in some rural areas of Suleimaniya governorate [9–11]. In 2018, the prevalence was 37.5%, ranging from 3.1% in Dohuk governorate to 45.1% and 50.1% in Suleimaniya and Erbil governorates [12]. Clitoridectomy, or Type I FGM, is the most common form of FGM practiced in the Iraqi Kurdistan Region [10].

### The Iraq Multiple Indicator Cluster Survey

The Iraq Multiple Indicator Cluster Survey (MICS) 2011 and 2018 are the latest two MICSs conducted in Iraq [12, 13]. The Iraqi Central Statistics Organization carried out these surveys in cooperation with the Kurdistan Regional Statistics Office, the United Nations Children's Fund (UNICEF), and the Ministry of Health of Iraq. The aim of MICS is to provide high quality data for assessing the situation of children, adolescents, women, and households for monitoring progress towards national goals. These two surveys covered all the 18 governorates in Iraq, including Dohuk, Erbil, and Suleimaniya governorates in the Iraqi Kurdistan Region. The 2011 and 2018 surveys adopted a two-stage sampling strategy. In the first stage, primary sampling units of each district were selected by systematic probability proportional to size. Then, these primary sampling units in each district were distributed between urban and rural areas proportionately. The 2011 survey included a representative sample of 11,384 women who answered a question on FGM status. The 2018 Iraq survey included a representative sample of 3117 women who answered a question on FGM status. Different questionnaires were used in the MICS 2011 and 2018 [14, 15]. The women's questionnaire targeted the women between 15 and 49 years in each household and collected data on woman's characteristics, including FGM status.

### Rational and purpose of the study

Since 2007, media and advocacy campaigns against FGM have been conducted in the Iraqi Kurdistan Region [16]. The Iraqi Kurdistan parliament issued the act of combating domestic violence in 2011, which included clauses that prohibit and criminalize FGM [17]. These efforts might have increased awareness and changed the attitude of the community about FGM. However, awareness about the practice and its harms might not be sufficient to eradicate a practice embedded deeply in the roots of society's culture [18]. Research has not adequately assessed the change in awareness about FGM as a result of these preventive measures. The impact of these preventive measures on the FGM rates and trend is also not well studied in Iraqi Kurdistan Region.

Several religious, social, and cultural factors are together responsible for practicing FGM in Iraqi Kurdistan Region. Research has shown that several factors can be associated with the increased prevalence of FGM, including the age of the girl or woman, education level of the girl and their parents, the economic status of the family, living in rural areas, and the FGM status of the mother [10, 19]. In 2011, the prevalence of FGM in Iraqi Kurdistan Region was lower in the younger age group (15–30 years) than the older one (31–49 years), which might indicate a decreasing trend of FGM [9]. The change in the trend and rates of FGM over time is not well studied in Iraqi Kurdistan Region, and the associated factors are not examined adequately. This study aimed to assess the trends and changes in the FGM rates in Iraqi Kurdistan Region between 2011 and 2018 and determine the associated factors.

## Methods

This study included secondary data analysis of the Iraq MICS 2011 and 2018 [12, 13]. UNICEF and its national partners conducted the primary data analysis of each of these two surveys. The main findings of this primary analysis were included in the main survey report for each survey. These reported findings included the number and frequency of FGM cases by governorates and other sociodemographic factors for the whole of Iraq.

In the MICS surveys, written or verbal consent was obtained for each participant. For children aged 15–17 years, parent or guardian's consent was obtained prior to the child’s assent. All participants were informed about the voluntary nature of participation and the anonymity and confidentiality of information. The participants were informed about the right to refuse to answer questions and stop the interview at any time.

The Research Ethics Committee of Hawler Medical University approved the current study, which involved secondary analysis of the two MICS surveys. UNICEF granted the author permission to access and use the datasets of both surveys. The datasets of the women aged 15–49 years from the three governorates of Iraqi Kurdistan Region who answered a question on their FGM status were initially separated from the rest of the datasets. Thus, the datasets of 11,384 women from the 2011 survey and 3117 women from the 2018 survey were used for the data analysis. The self reported FGM status of the interviewed women was obtained from the datasets and was used as the primary outcome variable. The age and education of the women, the economic situation of the household, and the governorate and area of residence were the exposure variables.

The Statistical Package for the Social Sciences (version 21) was used for data analyses. The prevalence and 95% confidence interval of FGM were calculated for both 2011 and 2018 by the governorates for the Iraqi Kurdistan Region. The trend of the determinants of FGM that included age, education level, economic status, and area of residence were compared over the study period from 2011 to 2018 using the Chi-square test. The prevalence of FGM and 95% confidence interval were calculated according to these determinants to assess the effect of the change in these determinants over the study period on the prevalence of FGM. The relative change in the prevalence or the trend of FGM between 2011 and 2018 was computed for each governorate and each of the main variables of age, education, wealth, and area of residence ((FGM prevalence 2018 − FGM prevalence 2011)/FGM prevalence 2011).

## Results

The prevalence of FGM in 2018 was high in Erbil and Suleimaniya governorates (50.1% and 45.1%) and relatively low in Dohuk governorate (3.1%). Compared to the prevalence in 2011, the prevalence of FGM decreased remarkably in all governorates of the Iraqi Kurdistan Region in 2018. The decrease in the prevalence of FGM was highest in Dohuk governorate, followed by Erbil and Suleimaniya governorates (relative change = −32.7%, − 20.3%, and − 19.3%, respectively). The decrease in the prevalence was statistically significant in Erbil and Suleimaniya. Details are shown in Table 1.

**Table 1:** Comparison of the prevalence of FGM by different governorates of Iraqi Kurdistan Region and the rest of Iraq between 2011 and 2018

Governorate	2011			2018			Relative change in %^a^
	Sample size	FGM		Sample size	FGM		
		No	% (95% CI)		No	% (95% CI)	
Dohuk	1807	83	4.6 (3.6–5.6)	873	27	3.1 (1.9–4.2)	− 32.7
Erbil	3686	2320	62.9 (61.4–64.5)	1079	541	50.1 (47.2–53.1)	− 20.3
Suleimaniya	5891	3290	55.8 (54.6–57.1)	1165	525	45.1 (42.2–47.9)	− 19.3

^a^FGM prevalence in 2018–FGM prevalence in 2011)/FGM prevalence in 2011

In 2018, the prevalence of FGM in Iraqi Kurdistan Region was higher among the older age groups, those with no or primary education, and those in the middle wealth group. The prevalence of FGM decreased remarkably and significantly from 2011 to 2018 in all age groups; such a decrease was highest in the younger age groups, particularly among those 20–24 years old and 15–19 years old (relative change =  −43.0% and − 39.2%, respectively). A statistically significant higher proportion of women had secondary and higher education in 2018 than in 2011 (57.2 vs. 36.1, *P* < 0.001). The prevalence of FGM decreased remarkably and significantly from 2011 to 2018 in all education groups, and the decrease was highest in those with secondary and higher education (relative change = −32%). A statistically significant higher proportion of women were in the wealthier economic groups in 2018 than in 2011. Regarding the economic situation, the prevalence of FGM decreased significantly from 2011 to 2018 in the poorest and second quintile, and the highest decrease was among these two groups (relative change = −38.8% and − 27.2%, respectively). The prevalence of FGM increased in the wealthiest group, with a relative change of 14.9%. The proportion of women living in urban areas significantly increased in 2018 compared to 2011 (82.7 vs. 66.3, *P* < 0.001). The prevalence of FGM was very similar in those living in urban and rural areas. Compared with 2011, the prevalence of FGM significantly decreased in both areas in 2018, and the decrease was higher in rural areas than urban areas (relative change = −35.3% and − 27.4%, respectively). Details are provided in Table 2.

**Table 2:** Comparison of the prevalence of FGM in Iraqi Kurdistan Region by different variables between 2011 and 2018

Variable	Sample size and prevalence of characteristic					FGM				
	2011		2018		*P* value	2011		2018		Relative change in %^a^
	No	%	No	%		No	% (95% CI)	No	% (95% CI)	
*Age*										
15–19	2176	(19.1)	523	(16.8)	< 0.001	807	37.1 (35.1–39.1)	118	22.6 (19.0–26.1)	− 39.2
20–24	2228	(19.6)	508	(16.3)		977	43.9 (41.8–45.9)	127	25.0 (21.2–28.8)	− 43
25–29	1977	(17.4)	469	(15.0)		1011	51.1 (48.9–53.3)	159	33.9 (29.6–38.2)	− 33.7
30–34	1634	(14.4)	513	(16.5)		906	55.4 (53.0–57.9)	192	37.4 (33.2–41.6)	− 32.5
35–39	1493	(13.1)	435	(14.0)		839	56.2 (53.7–58.7)	174	40.0 (35.4–44.6)	− 28.8
40–44	1090	(9.6)	385	(12.4)		650	59.6 (56.7–62.5)	182	47.3 (42.3–52.3)	− 20.7
45–49	786	(6.9)	284	(9.1)		503	64.0 (60.6–67.4)	141	49.6 (43.8–55.5)	− 22.4
*Education*										
None	3353	(29.5)	489	(15.7)	< 0.001	2079	62.0 (60.4–63.6)	243	49.7 (45.3–54.1)	− 19.9
Primary	3919	(34.4)	841	(27.0)		2031	51.8 (50.3–53.4)	382	45.4 (42.1–48.8)	− 12.4
Secondary +	4112	(36.1)	1787	(57.3)		1583	38.5 (37.0–40.0)	468	26.2 (24.2–28.2)	− 32
*Wealth index quintiles*										
Poorest	3276	(28.8)	91	(2.9)	< 0.001	2000	61.1 (59.4–62.7)	34	37.4 (27.4–47.3)	− 38.8
Second	3071	(27.0)	100	(3.2)		1603	52.2 (50.4–54.0)	38	38.0 (28.5–47.5)	− 27.2
Middle	2440	(21.4)	191	(6.1)		1171	48.0 (46.0–50.0)	83	43.5 (36.4–50.5)	− 9.5
Fourth	1541	(13.5)	450	(14.4)		608	39.5 (37.0–41.9)	165	36.7 (32.2–41.1)	− 7.1
Richest	1056	(9.3)	2285	(73.3)		311	29.5 (26.7–32.2)	773	33.8 (31.9–35.8)	14.9
**Area**										
Urban	7548	(66.3)	2577	(82.7)	< 0.001	3662	48.5 (47.4–49.6)	908	35.2 (33.4–37.1)	− 27.4
Rural	3836	(33.7)	540	(17.3)		2031	52.9 (51.4–54.5)	185	34.3 (30.3–38.3)	− 35.3

^a^(FGM prevalence in 2018 − FGM prevalence in 2011)/FGM prevalence in 2011

## Discussion

This study showed that the prevalence of FGM decreased remarkably and significantly from 2011 to 2018 in the Iraqi Kurdistan Region. However, the prevalence remained very high in 2018 in Erbil (50.1%) and Suleimaniya (45.1%) governorates. The trend of the prevalence of FGM showed a similar decline in most other countries during the last few decades. A study examined the trend of FGM in 22 countries over 30 years and included women born between 1965 and 2000 in each country showed that FGM had fallen over time in almost all countries examined. The decline in the included countries from the Middle East was relatively low, with a prevalence difference of − 7.2 in Egypt and − 7.3 in Yemen. Iraq and Iran were not included in that study [20]. Another study assessed the changing prevalence of FGM over time among girls aged 0–14 years in Africa and Western Asia between 1990 and 2017 revealed that the decline in the prevalence was highest in East Africa, followed by North and West Africa. However, the prevalence of FGM in Western Asia, including Iraq and Yemen, has gone up, rising by 1% in 1997 and 15.9% in 2013 [21]. It is not clear whether such an increase in Iraq and Yemen is a reverse trend or is related to the difference in the sampling strategy of the survey in these two periods. A reverse trend of FGM could be related to the prevailing risk factors in some countries, such as poverty, poor education, gendered cultural forces, and relating FGM to marriage [21].

There is evidence of a significant and continuous decline in the prevalence of FGM across countries and regions, particularly among children [21, 22]. Some studies have attributed this decline to the success of the national and international investment and policy intervention in the last few decades, including the legal ban, which is currently in place in most countries [21, 23]. Despite the decline of FGM practice globally, no significant changes in the prevalence are detected at the national level in many countries. Even within a country, mixed changes are evident at the regional and individual levels [24]. The area of residence and the age are considered significant determinants for such difference in the practice, which suggests that community factors rather than individual factors play an important role in the trend of the practice. The presence of such interdependent determinants and expectations about the practice is supportive of the role of social norms and convention theories in the decline or rise of FGM practice [24, 25].

In the current study, the prevalence of FGM in 2018 was remarkably lower in the younger age girls and women, and the decrease in prevalence compared to 2011 was more evident among this group. This might indicate a continuous reduction in the practice over time in this region. Such a trend is promising and predicts even lower rates in the future. The reduction in the practice and the decrease in the prevalence of FGM might be related to the increased peoples' awareness about FGM, particularly about the health consequences of FGM. The effectiveness of educational programs that emphasize the negative health consequences of FGM on changing the attitude toward FGM is well documented [26]. It is not clear if the decline in FGM level revealed in the current study or any increased peoples' awareness is directly related to the preventive measures applied in the region, such as awareness campaigns and the legislation banning the practice. Reports of the high prevalence of FGM in the Iraqi Kurdistan Region in the late 2000s have resulted in the launch of the “Stop FGM in Kurdistan” campaign by a number of civil society organizations and women's rights groups [16]. This awareness-raising and advocacy campaign and the extensive efforts made in the Iraqi Kurdistan Region to abandon FGM practice helped in passing the act of combating domestic violence in June 2011 [17]. This law includes a number of provisions that criminalize FGM in Iraqi Kurdistan Region. Civil society organizations and some religious leaders have been actively engaged in helping to prevent and combat FGM. Religious obligation is an important reason for practicing FGM in many countries, including Iraqi Kurdistan Region [10, 11]. As FGM is falsely linked to religion, the role of religious leaders has been very important in raising the awareness of the people against this harmful practice and in de-linking it from religion [27]. These initiatives could have contributed to increased awareness of the health risks of FGM in the new generation [10].

The decline in the FGM level could also be related to having an increasingly larger number of educated people, particularly women, in society [28]. Having a higher reduction among those with secondary and higher education in the current study supports this explanation. With the increasing globalization, cultural diversities, and media effect, societies have developed a greater awareness of different health issues, including harmful cultural practices and behaviors such as FGM, child marriage, and forced marriage [29, 30]. Similar to the current study, another study from Egypt suggested that increases in women’s education might be causally related to reducing the prevalence of FGM [31].

The current study showed that the highest decline in FGM prevalence was in the poorest and lowest economic groups. A previous study from the Iraqi Kurdistan Region showed that a lower wealth index of the household was significantly associated with a higher occurrence of FGM in daughters [19]. Another study, including seven African countries, showed that adolescent girls with a lower economic status had greater support to FGM than the wealthiest group in six out of the seven countries [32]. This promising finding in the current study might be related to having better access to education and awareness by the poor groups [28].

As shown in the current study, the increase in FGM prevalence in the wealthiest group is not well understood. Research from other settings has shown that women living in wealthier households are more likely to oppose FGM [33]. The economic situation in Kurdistan Region witnessed many ups and downs over the last decades. This might have changed the society by having many poorly educated and those who still abide by cultural traditions becoming wealthier. On the other hand, the economic hardship and the cuts and delays in public employees' salary have shifted many better educated to less wealthy groups.

The increase in the FGM prevalence among the wealthiest groups might also be related to the medicalization of FGM. With the greater access to healthcare services and increased awareness of the adverse health consequences of FGM, health professionals have become increasingly involved in performing FGM [34]. People with more financial ability might choose to medicalize FGM thinking that they avoid adverse health effects. In Iraqi Kurdistan Region, FGM is primarily performed by traditional birth attendants and traditional circumcisers. A study in 2009 found that health care professionals are responsible for performing only around 1% of procedures [10]. No new data or studies are available about the medicalization of FGM in Iraqi Kurdistan Region. Although the medicalization of FGM is prohibited by law in the region, the rapid growth of the poorly controlled private health sector [35] might have resulted in increased FGM medicalization in this region.

The prevalence of FGM in 2018 was very similar in urban and rural areas, but the decline from 2011 to 2018 was higher in the rural areas. Our findings agree with Abdulah et al., who refuted the previous claims of having a higher prevalence of FGM in rural areas than in urban areas of Iraqi Kurdistan Region [36]. The higher decline of FGM in rural areas revealed in the current study could be related to rural–urban migration in addition to having more access to education, awareness, and technology in rural areas. Moreover, the rural areas in Iraqi Kurdistan Region are slowly changing to more suburban and urban areas [37]. Unlike our study, another study showed that FGM is declining more rapidly in urban areas at the global level [38].

The current study provides an understanding of the trend and changes in the prevalence of FGM between two times periods. It provides the analysis at the governorates' level and according to some associated factors. However, it does not look at the actual drivers or reasons leading to the changes in the prevalence or the practice of FGM in Iraqi Kurdistan Region. Studies at the individual or household levels are needed to understand these factors in an in-depth manner.

## Conclusion

The trend of FGM in Iraqi Kurdistan Region remarkably and significantly declined from 2011 to 2018. Further decline is predicted in the near future due to having lower rates and a high decline in girls and women of the younger age groups. However, the rates remained high in Erbil and Suleimaniya governorates that need intensifying the preventive measures. The education level of women seems to play a primary role in the declining trend and should be considered in future efforts to ban the practice in Iraqi Kurdistan Region. Improving women’s education and literacy and empowering them with more health education and awareness about FGM consequences can help in combating the FGM practice and further reducing its prevalence and health and social adverse effects. Intervention should not only focus on women, but it should also target men and other community leaders and actors that might be part of decision making and can play important roles in prevention efforts. Future in-depth studies are needed to explore and understand the reasons for the increased prevalence in wealthier groups.

## Data Availability

The datasets used for the current study are available on the Multiple Indicator Cluster Survey website (https://mics.unicef.org/) and accessible on reasonable request.

## Abbreviation

FGM: Female genital mutilation
